# Unique Structural Features Facilitate Lizard Tail Autotomy

**DOI:** 10.1371/journal.pone.0051803

**Published:** 2012-12-19

**Authors:** Kristian W. Sanggaard, Carl Chr. Danielsen, Lise Wogensen, Mads S. Vinding, Louise M. Rydtoft, Martin B. Mortensen, Henrik Karring, Niels Chr. Nielsen, Tobias Wang, Ida B. Thøgersen, Jan J. Enghild

**Affiliations:** 1 Department of Molecular Biology and Genetics, Center for Insoluble Protein Structures (inSPIN), Interdiciplinary Nanoscience Center (iNANO), Aarhus University, Aarhus, Denmark; 2 Department of Biomedicine, Aarhus University, Aarhus, Denmark; 3 Department of Clinical Medicine - Research Laboratory for Biochemical Pathology, Aarhus University, Aarhus, Denmark; 4 Department of Chemistry, Center for Insoluble Protein Structures (inSPIN), Interdisciplinary Nanoscience Center (iNANO), Aarhus University, Aarhus, Denmark; 5 Department of Clinical Medicine - Center for Functionally Integrative Neuroscience, Aarhus University, Aarhus, Denmark; 6 Department of Clinical Medicine - The Department of Thoracic and Cardiovascular Surgery T, Aarhus University, Aarhus, Denmark; 7 Institute of Chemical Engineering, Biotechnology and Environmental Technology, Faculty of Engineering, University of Southern Denmark, Odense, Denmark; 8 Department of Bioscience – Zoophysiology, Aarhus University, Aarhus, Denmark; Kaohsiung Chang Gung Memorial Hospital, Taiwan

## Abstract

Autotomy refers to the voluntary shedding of a body part; a renowned example is tail loss among lizards as a response to attempted predation. Although many aspects of lizard tail autotomy have been studied, the detailed morphology and mechanism remains unclear. In the present study, we showed that tail shedding by the Tokay gecko (*Gekko gecko*) and the associated extracellular matrix (ECM) rupture were independent of proteolysis. Instead, lizard caudal autotomy relied on biological adhesion facilitated by surface microstructures. Results based on bio-imaging techniques demonstrated that the tail of *Gekko gecko* was pre-severed at distinct sites and that its structural integrity depended on the adhesion between these segments.

## Introduction

Tail autotomy has evolved independently in vertebrates and invertebrates [1] but is particularly common in lizards. Among vertebrates, the tail is the only appendage that can be lost by voluntary shedding [2], which functions as an anti-predator strategy. The detached tail continues to wiggle by anaerobic metabolism for up to 30 minutes, thereby distracting the attention of the predator from the escaping lizard [1], [3]. Although autotomy might save the life of the lizard, there is a significant cost to the animal due to the vital functions of the tail for movement, establishing social status, and as a fat-storage organ [4]. Indeed, some lizard species, that use their tail for fat storage, return to site where the tail was lost, and eat the autotomized tail most likely to compensate for the loss of a major fat reserve [4]. The fracture usually occurs just in front of the segment where the lizard was grabbed, or no more than 3 fracture plans anterior to this, supporting that autotomy is associated with a significant cost for the lizard [5].

Tail autotomy occurs at preformed horizontal fracture planes. These can be either intravertebral, where the fracture plane crosses the caudal vertebra, or intervertebral, which is a less common form of autotomy [6]. During intravertebral tail autotomy, the fracture occurs at a distinct preformed area of weakness that follows the myoseptum to separate the adjacent segments of the axial musculature [7]. The lizard assists the autotomy by contracting muscles around the fracture planes, as demonstrated by the larger force required to detach the tail in euthanised or unconscious lizards (i.e., “passive shedding”) [7]. These muscle contractions are also likely to facilitate splitting of the skin and muscles to complete the release of the tail [8]. Initially, we speculated that proteolysis in concert with the apparent muscle activity facilitated tail autotomy. The involved proteases would likely utilise a novel proteolytic mechanism because triple-helical collagen is highly resistant to proteolysis and is only cleaved, albeit slowly, by matrix metalloproteases (MMPs). The turnover rates (k*cat*) of MMP-1 are only 53.4 h^−1^ (human type I collagen), 1.0 h^−1^ (human type II collagen) and 565 h^−1^ (human type III collagen) [9], thereby demonstrating that classical MMP degradation is too slow to explain the rapid release of the tail.

In the present study, we have used proteomics, and a combination of bio-imaging methods to investigate the mechanism of tail autotomy in Tokay gecko. We show that (i), the process is protease-independent (ii), the pre-formed “score lines” in the fracture planes are maintained by adhesion and (iii), microstructures at the terminal end of the muscle fibers most likely are involved in the release the tail.

## Materials and Methods

### Materials

An Ettan CAF™ MALDI sequencing kit was obtained from GE Healthcare. ZipTip_C18_ pipette tips were from Millipore. MS-grade trypsin was from Promega. Glu-fibrinopeptide B, 4-sulfophenyl-isothiocyanate (SPITC), and α-cyano-4-hydroxycinnamic acid were obtained from Sigma-Aldrich.

### Animals

*Gekko gecko* were obtained from commercial suppliers and housed in terrariums in a heated room (26°C) at Aarhus University. The animals were fed insects several times per week, and fresh water was always available. Tails were amputated by induced autotomy. To a large extent, the fracture plane used for shedding was dictated by controlling the positioning of the bilateral pressure that was applied. The desired fracture plane depended on the specific type of analysis. For macroscopic photography and histochemical analyses of the tail, an animal was euthanised by an overdose of the pentobarbital (Nembutral 50 mg kg^−1^, i.m.), and the tail was removed post-mortem by induced autotomy (“passive shedding”).

### Ethics Statement

Animal experiments were conducted in accordance with permission from the Danish Inspectorate for Animal Experiments.

### Gel Electrophoresis, Mass Spectrometry and BLAST Analyses

Immediately after tail shedding, the moisture at the exposed fracture of the casted tail stump was absorbed using filter paper. The paper was boiled for 5 min in SDS sample buffer containing 30 mM dithiothreitol and was then centrifuged. Subsequently, the supernatant was subjected to SDS-PAGE using 5–15% gradient gels with a glycine/2-amino-2-methyl-1,3-propanediol HCl buffer system [10]. The resolved proteins were visualised by silver staining [11]. All distinct bands were excised from the gel, and in-gel digestion with trypsin was performed as previously described [11], except that the reduction with dithiothreitol and carboxymethylation with iodoacetamide were omitted. The resulting peptides were either: i) purified directly, ii) labelled with SPITC [12] before purification, or iii) purified and prior to the elution modified on the resin according to the Ettan CAF™ MALDI sequencing kit protocol. The peptide purification was performed using ZipTip_C18_ pipetting tips as recommended by the manufacturer. The purified peptides were eluted directly onto MALDI target-plates using 1 µL of matrix solution containing 70% (v/v) acetonitrile, 0.03% trifluoroacetic acid (v/v), and 0.4% (w/v) recrystallised α-cyano-4-hydroxycinnamic acid. The eluted peptides were analysed in a Q-TOF Ultima mass spectrometer (Waters/Micromass) calibrated prior to the analyses using a polyethylene glycol mixture (from *m/z* 50 to *m/z* 3000). Each MS spectrum was externally calibrated using Glu-fibrinopeptide B (*m/z* 1570.6774). Data processing of the obtained MS and MS/MS data was performed using Masslynx MS software (Waters/Micromass). The PepSeq-software of the Masslynx MS software package facilitated the manual interpretation of the MS/MS spectra. The obtained peptide sequences were subsequently analysed using MS-BLAST (http://genetics.bwh.harvard.edu/msblast/) [13]. Only positive hits obtained by the MS-BLAST software were accepted.

### Magnetic Resonance Imaging

The released tail was immersed in 4% buffered formalin (Lillie’s fixative) for 24 h and was then transferred to 45 ml phosphate buffered saline for 12 h. Subsequently, the tail was placed in a 10 mm nuclear magnetic resonance tube, where it was suspended in fluorinert (FC-43) to suppress background signal. Imaging was performed on a vertical 54 mm-bore magnet (Bruker Advanced II, BioSpin GmbH, Rheinstetten, Germany) with a field strength of 16.4 T. All magnetic resonance experiments were performed using a MicroImaging 5 probe (max. gradient strength of 1.445 T/m in 110 µs), a Great 60 Imaging system, a saddle coil with inner diameter of 10 mm, and a gradient cooling temperature of 20°C. Images were acquired with a 3D Fast Low-Angle Shot (FLASH) sequence using the following parameters: echo times/repetition times = 6.78 ms/17.93 ms or 20.19 ms, 75 averages, 15° flip angle, and a 1×1×1 cm field of view. The acquisition matrix of 400×512×128 was zerofilled to 512×512×128 to yield a spatial resolution of 19.5 µm×19.5 µm×78.1 µm. Post-processing was performed using MATLAB R2010a (The MathWorks, Natick, MA, USA) prior to 3D rendering employing OsiriX Imaging Software [14], [15].

### Scanning Electron Microscopy (SEM)

Immediately after autotomy, the casted tail stump was immersion-fixed in 4% buffered formalin (Lillie’s fixative) for 24 h at room temperature followed by 1–4 days at 4°C. The stump was then rinsed extensively with 50 mM Tris-HCl, pH 7.4. Specimens were post-fixed at room temperature for 2–3 h, first with 1% tannic acid/H_2_O and then with 1% OsO_4_/H_2_O. Subsequently, the specimens were rinsed in distilled water for several hours before dehydration through graded concentrations of ethanol and were preserved by freeze drying/CPD. The specimens were mounted on an aluminium stub, Pt-coated and observed in a FEI NOVA NanoSEM 600.

### Microscopy

The casted tail stump was fixed in 4% buffered formalin and subsequently divided longitudinally and embedded in paraffin. Sections of 3 to 5 µm thickness were cut and stained with Mallory’s trichrome staining solution to evaluate the differentiation of connective tissue elements. Areas in which the ‘cellular zipper’ was identified by light microscopy were punched, embedded in epon and processed for electron microscopy (EM) according to standard methods. Tail fragments from a separate animal were immersed in Tyrode’s buffer containing 1% glutaraldehyde and 3% paraformaldehyde, embedded directly in epon and processed for EM according to standard methods. It should be noted that no difference was observed between the two methods.

## Results and Discussion

**Figure 1:**
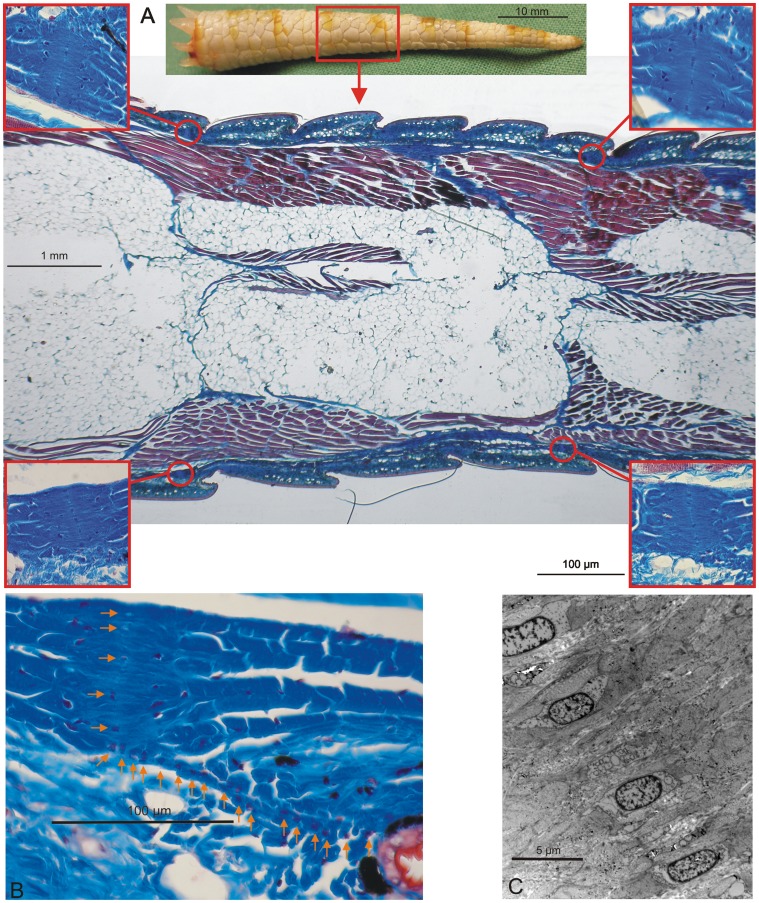
The gecko tail is segmental in nature. A) A histochemical analysis of a longitudinal *Gekko Gecko* tail section is shown (Mallory’s trichrome stain). The connective tissues and scales appear blue, the muscles appear red, and the adipose tissues appear white. The data illustrate the segmented structure of the gecko tail. The inserts focus on the areas where the collagen fibers are condensed. **B)** A histochemical analysis focusing on the proposed autotomy septum in the dermis. The arrows show the position of the septum in the condensed collagen area. In addition, arrows indicate the fracture plane from the condensed collagen area to the indentation between two scales where the severing takes place. **C)** Electron microscopy analysis focusing on the proposed autotomy septum in the condensed collagen area. The analysis reveals the presence of embedded cells in the proposed fracture plane that may function as a “cellular zipper”.

**Figure 2:**
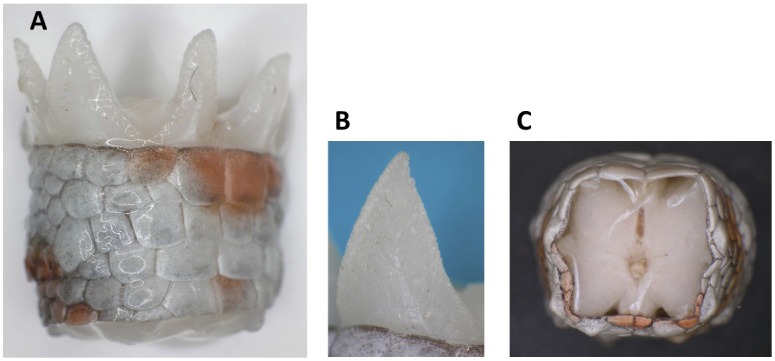
Wedge-shaped extensions project from the proximal end of the released tail stump. A) The proximal part of a released tail stump is observed from the side. The picture shows the wedge-shaped extensions. **B)** A wedge-shaped extension. **C)** The distal part of the tail that remains on the animal. The grooves, in which the extensions are fitted, in an end-to-end tapered “finger joint” fashion, are only vaguely seen just below the dermis. Immediately after autotomy, the skin contracts around the end of the tail stub, making the grooves difficult to observe.

Passive shedding was demonstrated by placing euthanised *Gekko gecko* at 5°C for 24 hours followed by severing elicited at different fracture planes to illustrate the segmented and “precut” nature of the tail (Fig. S1) [16]. The “precut” architecture of the tail was further supported by histochemical analyses (longitudinal section) revealing the individual segments of the tail (Fig. 1A). In addition, analysis revealed the fat layer surrounding the vertebra and the muscle positioned between the dermis and the fat layer. The autotomy septum divided both the fat and muscle regions across the tail in each fracture plane, and in the longitudinal direction, a dorsal median septum, a ventral median septum, and a horizontal septum divided the tail into 4 individual longitudinal segments. Each of the 4 muscle segments formed two wedge-shaped extensions projecting from the proximal end of the released portion of the tail (Fig. 2A and 2B). Prior to autotomy, these extensions assembled the tail in an end-to-end tapered “finger joint” fashion because they fit grooves in the distal part of the tail stump. These grooves are difficult to observe after autotomy because the skin contracts around the end of the tail stub (Fig. 2C). After detachment, the autotomy septum adhered to the tail stub, likely as a defensive mechanism to protect the exposed part of the tail [5]. The autotomy septum can be observed between the fat and muscle segments but was somewhat obscured in the dermis, where it appears to merge with the dermal layers [5]. A more detailed histochemical analysis of the area showed that at intervals corresponding to the distance between fracture planes, the collagen layer was thickened and condensed (Fig. 1). The position of these condensed areas suggests that they represent the autotomy septum through the dermis layer. Cells oriented in a radial fashion were observed running through the collagen condensation and continuing through the dermis towards an epidermal indentation between adjacent scales (Fig. 1). This chain of cells may represent the dermis fracture plane and could function as a “cellular zipper”. Similar structures have been observed in sea lilies (crinoidea) [17], where they are designated juxtaligamental cells [18]. During sea lily autotomy, the juxtaligamental cells apparently discharge material to facilitate the rapid loss of tensile strength in the fracture plane [18], [19]. Likewise, the observed *Gekko gecko* cells (Fig. 1C) contain numerous granules [17]. The collagen structure in the fracture planes of sea lilies is referred to as mutable collageneous tissue, or “smart” collagen, due to the capacity to undergo a rapid change of mechanical properties [20], [21]. The anatomical similarity of the fracture sites of sea lilies and geckos suggests common autotomy features in the dermis of the *Gekko gecko* and that of echinoderms. However, the “cellular zipper” constitutes only a small part of the fracture plane, and “smart” collagen is likely to play a minor role in the overall mechanism of *Gekko gecko* autotomy.

**Figure 3:**
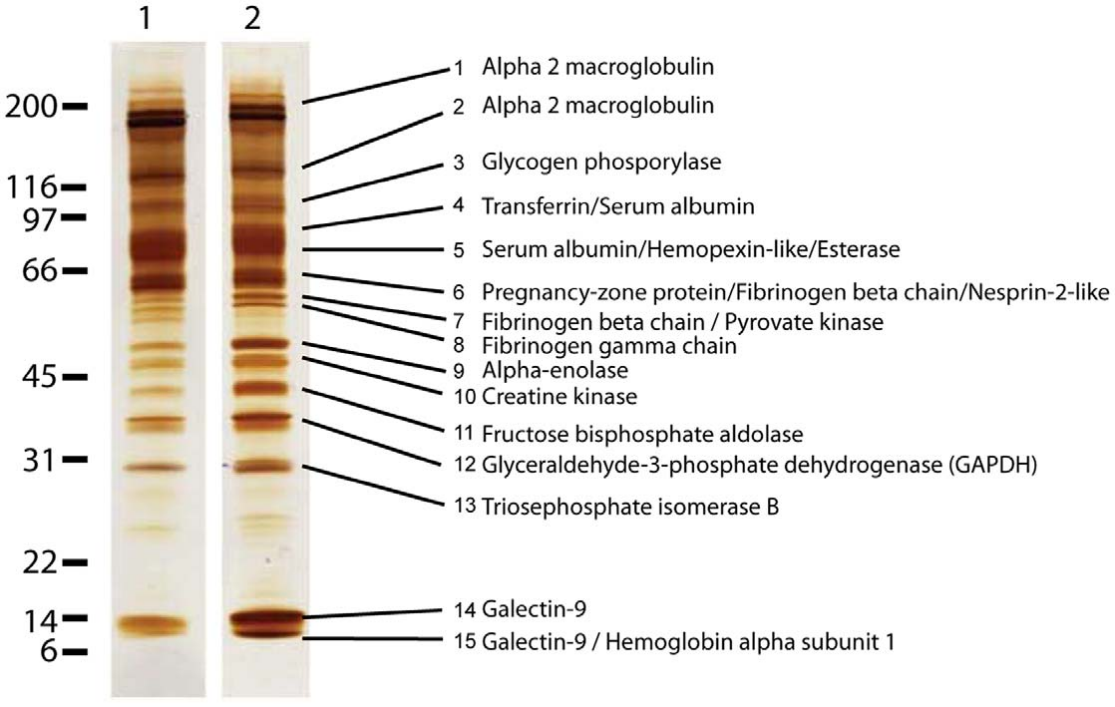
Proteolytic enzymes are conspicuously absent from the fracture planes. The proteins in the liquid from the proximal (lane 1) and the distal (lane 2) part of the fracture plane were separated by SDS-PAGE analysis and subsequently silver-stained. All visible bands were subsequently excised, subjected to *de novo* sequencing using mass spectrometry, and the proteins present in the different bands were then identified based on homology analyses (Table S1). In total, 18 proteins were identified in the liquid, and one of the dominant groups of proteins was cytosolic proteins involved in glycolysis. No putative proteolytic enzymes were present, suggesting that proteolysis is not involved in tail autotomy.

The proteins released during autotomy were collected by sampling the clear liquid observed on the tail stubs. These proteins could possibly provide clues to the autotomy mechanism, and SDS polyacrylamide gel electrophoresis analysis demonstrated that the protein compositions of the proximal and the distal parts of the tail were highly similar (Fig. 3). The observed protein bands were excised and digested with trypsin, and selected peptides were sequenced by *de novo* tandem mass spectrometry [22]. The deduced amino acid sequences were used to query databases of protein sequences to identify homologous proteins (Table S1). The only identified protein that potentially is directly involved in the autotomy process is the identified esterase. A BLAST search with the identified esterase (XP_002415879) identifies the bovine variant as the closest homolog at the protein level. This bovine homolog terminates signal transduction at the neuromuscular junction by rapid hydrolysis of the acetylcholine released into the synaptic cleft (P23795, NCBI). It is possible that the identified esterase serve a similar function in *Gekko gecko* during tail autotomy.

Although all apparent protein bands were identified, endogenous proteolytic enzymes were conspicuously absent. We cannot exclude the involvement of proteolytic enzymes during the detachment of the dermis, veins, or nerves. However, because proteolytic enzymes or proteolytic fragments were absent, the overall mechanism seems to be protease-independent, as supported by the known slow degradation of interstitial collagens [9]. Further examination of the determined protein profile did not provide additional clues because the known functions of the identified proteins were inconsistent with plausible tail autotomy mechanisms. In addition to plasma proteins, the dominant group of identified proteins was cytosolic and involved in glycolysis. Glycolytic enzymes are known to readily diffuse from vertebrate muscle fiber segments [23]. Therefore, our findings do not necessarily indicate that cell rupture takes place during tail autotomy. Various plasma proteins were detected, but they did not dominate the sample, which is in agreement with previous results suggesting that blood loss following autotomy is reduced by different strategies (e.g., sphincters and valves) [7].

**Figure 4:**
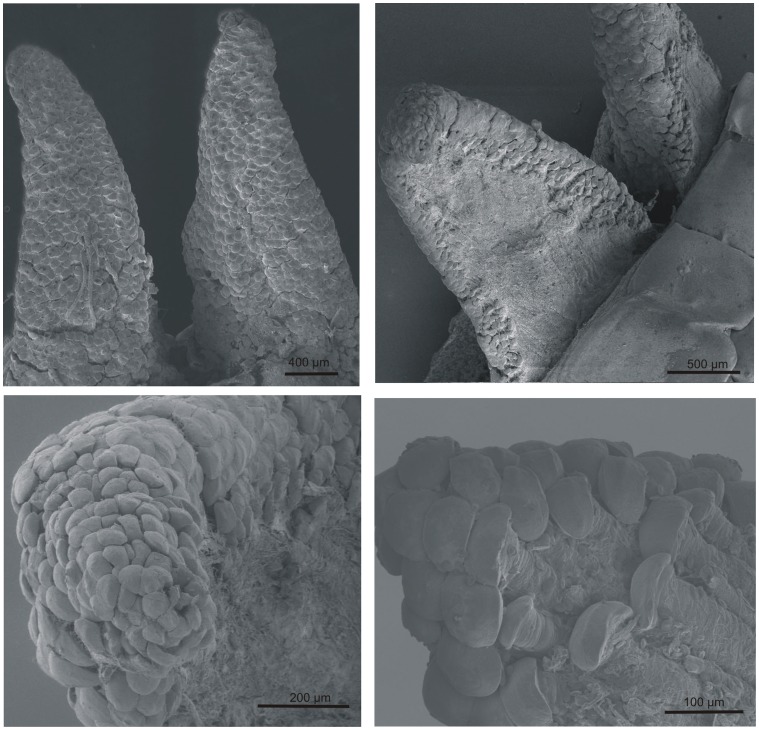
The muscle fibres terminate in “mushroom-shaped” structures. Scanning electron microscopy analyses of the wedge-shaped extensions (cranial margins of individual tail muscles) projecting from the proximal end of the released tail stump demonstrated the presence of “mushroom-shaped” structures at the termini of the muscle fibres after autotomy. These structures are present on all sides of the extensions except on the outer part.

**Figure 5:**
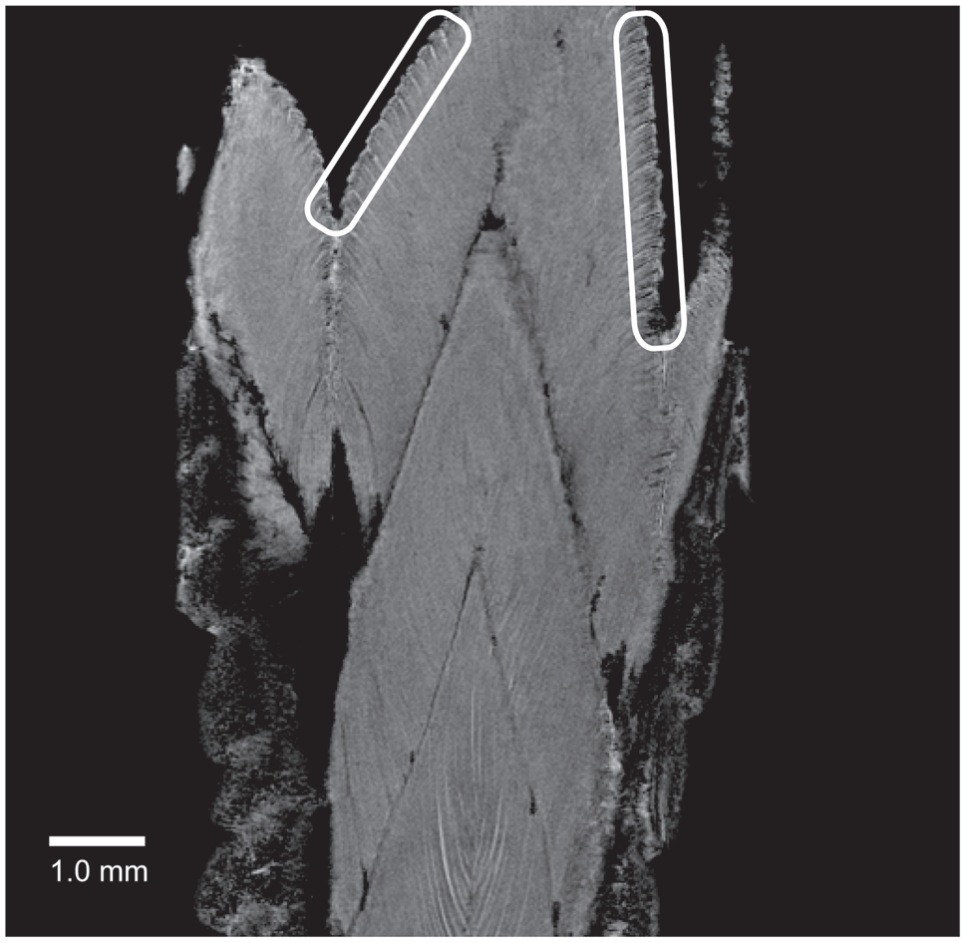
Cross-fracture-plane structures are absent in the gecko tail. Magnetic resonance imaging was used to analyse a detached tail stump (see also Movies S1–S3). The data revealed the structures of the muscle fibers in the different segments of the gecko tail. Two areas have been encircled to point out the position of the muscle fiber termini neighbouring the autotomy fracture plane. The image illustrates the expanded nature of these termini. The presence of these “mushroom-shaped” structures in the fracture plane and the absence of these structures in the interior muscle fibers illustrate the conformational change of the muscle termini that occurs during autotomy. The interdigitating arrangement of the muscle fibers are observable and the picture also shows that no through-going structures between muscle segments are observable, thereby supporting the concept that tail segments interact by adhesion forces.

The passive shedding capacity, precut segmented nature of the tail, and lack of proteases suggested that the structural integrity was mediated by surface interactions. The surface topography of the largest interacting surface consisted of wedge-shaped muscle extensions, thus warranting a more detailed examination of their surface properties. The surface topography was analyzed by scanning electron microscopy of the proximal surface of the shed tail stump. It demonstrated the presence of “mushroom-shaped” structures with diameters of 30–80 µm at the cranial margins of the muscle termini (Fig. 4). The exterior parts of the muscles running parallel to the dermis did not contain these structures, thereby reinforcing a role in the mechanism of autotomy (Fig. 2 and Fig. 4). To characterize the surface of the wedge-shaped muscle extensions in the fracture plane further, we analyzed a shed tail stump using magnetic resonance imaging (MRI) (Fig. 5 and Movies S1–S3). Using this approach, we were able to detect both the muscle fiber terminations in the fracture plane and the muscle fiber interactions in an “intact” fracture plane. The data illustrated the interdigitation arrangement of muscles in the tail rather than a simple “end-to-end” arrangement. This design is likely to facilitate adhesion by generating a larger surface area of interaction between two successive segments. In addition, the data did not supply evidence for structures going through the fracture plane. It further supports that biological adhesion is the main mediator of contact between tail segments (Fig. 5).

The muscle termini most likely adhere to the connective tissue septum in the fracture plane and divide the interacting surface into finer segments. Even though the “mushroom-shaped” structures at the terminal of the muscle fibers are most conveniently observed after autotomy, they are likely to be present in the intact tail. However, the structures are likely to have a more compact rod anatomy before autotomy, which may explains why they did not show up in MRI analysis (Fig. 5). Synthetic microfibers of a similar size as the “mushroom-shaped” structures have been shown to possess adhesion properties [24], [25] supporting that adhesive force may be formed between the muscle termini and the connective tissue septum. Consequently, both the numerous termini of the muscle fibers and the interdigitation of successive tail-segments are consistent with adhesion as the main mediator of contact between successive segments in the tail.

The observation that the “mushroom-shaped” structures are not visible in the MRI scans of the intact tail suggests that the characteristic morphology is caused by structural changes that expand the termini during tail loss. It is likely that the muscle termini in the intact tail have a flat surface, which facilitate adhesion to the fracture plane septum. During autotomy the termini probably expand and adopt the rounded appearance of the “mushroom-shaped” structures. Consequently, the interaction area, and thereby the adhesion, is lowered, facilitating autotomy. This result is in agreement with the hypothesis that these structures facilitate autotomy by lowering the friction between the interdigitating muscle structures in the tail during the autotomy process.

The climbing capacity of *Gekko gecko* also depends on adhesive forces; in this context, the adhesion is facilitated by hundreds of thousands of foot-hairs (setae) [26]. The adhesion by the spatula-formed setae on the toe pads of the lizard is flexible and temporary in nature. In contrast, the muscle termini attachment in the caudal fracture plane accounts for a more stable connection [27], [28] to limit the risk of accidental tail loss. Furthermore, the muscle termini lack hairs and are significantly larger (30–80 µm in diameter after autotomy) (Fig. 4) than the tips of the foot-hairs (0.2 µm). It also implies that a different adhesion strategy is exploited in the tail.

Based on the literature and the results present in the current paper the mechanism of lizard tail autotomy is likely to include the following events: i) Physical fixation of the tail by a predator, ii) nerve activation of the “voluntarily” tail shedding mechanism, and iii) muscle contraction and disruption of adhesion in the fracture plane facilitating autotomy. In addition, secretory products from the “cellular zipper” region is probably involved in the autotomy process in the collagen layer, similar to the observed autotomy process in echinoderms [17].

### Conclusion

Our data suggest that caudal autotomy in lizards is a biological friction- and adhesion-based phenomenon. The tail contains “score lines” at distinct horizontal fracture planes where the tail may be released as a response to predation. These scores penetrate all the way through the tissue where the structural integrity is maintained by adhesion forces. The interdigitation arrangement of muscles is likely to facilitate adhesion by generating a larger surface area of interaction. This architecture increases the contact area and most likely decreases the risk of accidental tail loss as opposed to a simple “end-to-end” arrangement. “Mushroom-shaped” microstructures at the muscle termini are observed after autotomy. These structures are likely to facilitate tail release by reducing the adhesion between tail segments. Furthermore, the segmentation permits the release in an orchestrated manner, a design that facilitates the lizard’s ability to shed its tail easy and quickly without employing a slow protease-based degradation of connective tissue.

## Supporting Information

Figure S1 **The tail of** ***Gekko Gecko*** **has a segmented nature with numerous fracture planes.** A *Gekko Gecko* was euthanized and placed at 5°C for 24 h. Subsequently, severing was elicited at different fracture planes to illustrate the segmented nature of the tail. Panel **A)** shows the intact animal with the tail broken at different fracture planes, and **B)** shows a close-up photo of the most proximal regions of the different generated tail segments.
(EPS)

Table S1 ***De novo*** **sequencing- and homology-based proteomics. A)** The band number (#) refers to the bands visualized by silver staining in the gel shown in Figure 3. The bands were excised and treated with trypsin, and the resulting peptides were analyzed by MALDI-MS/MS. From each band, all ions with the required intensity were sequenced. **B)** The sequenced peptides from each band are labeled with small letters. **C)** The m/z value of the observed precursor ions. **D)** The obtained MS/MS spectra were used to call the sequence using *de novo* sequencing techniques. “L” stands for the isobaric residues Leu or Ile; “X” represents any amino acid residue; “B” represents a putative trypsin cleavage site. The deduced peptide sequences were used to query the nr95 database using MS-BLAST, using the “L”, “X”, and “B” nomenclature. Only relevant entries that were defined as “positive hits” according to the MS-BLAST scoring system were reported. **E)** The proteins identified. **F)** The species and the accession number of the identified protein (based on the peptide hits in column E). Many of the identified proteins are from evolutionarily closely related species, such as *Xenopus Laevis* and *Zaocys dhumnades,* which support the identification of the homologous protein. **G)** The MS-BLAST score for the identified protein (column F). **H)** The common names of the identified proteins (these names are also used in column E). Some of the peptides used for the homology searches displayed homology with proteins that are homologous to the top-hit protein. The names of these homologous proteins are also listed in this column.
(PDF)

Movie S1 **Bridging structures are not present between tail segments.** The movie shows a sequence of sagittal images obtained by a 3D Fast Low-Angle Shot magnetic resonance sequence with fat suppression that progresses through the proximal end of the discarded Gecko tail. It demonstrates the interdigitating arrangement of muscle fibers and supports that fracture plane-crossing structures are absent. In addition, it visualizes the nature of the muscle termini, both in the fracture plane and in the intact tail (see also Fig. 5).
(MOV)

Movie S2 **The muscle fibres in successive tail segments display an interdigitated structure.** The movie shows a sequence of axial images obtained by a 3D Fast Low-Angle Shot magnetic resonance sequence with fat suppression that progresses through the discarded Gecko tail. The movie shows the peripheral position of the muscle fibres and demonstrates the interdigitated structure.
(MOV)

Movie S3 **3D-volume reconstruction of the proximal end of the discarded Gecko tail.** The movie is rendered from the sagittal images in Movie S1.
(MOV)
