# Supplementary material for: Unique Structural Features Facilitate Lizard Tail Autotomy
Source: PLoS One. 2012 Dec 19;7(12):e51803. doi: 10.1371/journal.pone.0051803 (PMC3526639; doi:10.1371/journal.pone.0051803)
Supplement: Table S1 — De novo sequencing- and homology-based proteomics. A) The band number (#) refers to the bands visualized by silver staining in the gel shown in Figure 3. The bands were excised and treated with trypsin, and the resulting peptides were analyzed by MALDI-MS/MS. From each band, all ions with the required intensity were sequenced. B) The sequenced peptides from each band are labeled with small letters. C) The m/z value of the observed precursor ions. D) The obtained MS/MS spectra were used to call the sequence using de novo sequencing techniques. “L” stands for the isobaric residues Leu or Ile; “X” represents any amino acid residue; “B” represents a putative trypsin cleavage site. The deduced peptide sequences were used to query the nr95 database using MS-BLAST, using the “L”, “X”, and “B” nomenclature. Only relevant entries that were defined as “positive hits” according to the MS-BLAST scoring system were reported. E) The proteins identified. F) The species and the accession number of the identified protein (based on the peptide hits in column E). Many of the identified proteins are from evolutionarily closely related species, such as Xenopus Laevis and Zaocys dhumnades, which support the identification of the homologous protein. G) The MS-BLAST score for the identified protein (column F). H) The common names of the identified proteins (these names are also used in column E). Some of the peptides used for the homology searches displayed homology with proteins that are homologous to the top-hit protein. The names of these homologous proteins are also listed in this column. (PDF) [file pone.0051803.s002.pdf]

Table S1

A	B	C	D	E	F	G	H
Band #	Peptide #	MH+ (observed)	Deduced peptide sequences	Peptide hits	Positive hits (accession and species)	Total score MS-BLAST	Common name and homologous proteins ("/") identified as positive hits
1	a)	2688.42	BLVLQCGXXAGVPGEFSTEVAGEG	Alpha 2 macroglobulin,	ref|XP_002190183.1 [Taeniopygia guttata]	183	alpha 2 macroglobulin
	b)	2241.22	TEGLPGSNTHLQLA	Alpha 2 macroglobulin			
	c)	1798.93	BLLQWNDVQLQDGLTR	no homologous			
	d)	1422.8	LLSGNDVQHHF	no homologous			
	e)	1226.64	BALGYLVS	Alpha 2 macroglobulin			
2	a)	1798.92	BLLQWNDVQLQDGLTR	Alpha 2 macroglobulin	ref|XP_002190183.1 [Taeniopygia guttata]	170	alpha 2 macroglobulin
	b)	1386.72	BVLEEGTRELQGR	no homologous			
	c)	1353.76	TEGLPGSNTHLQL	Alpha 2 macroglobulin			
	d)	1334.72	BFPLVYLQDPSR	Alpha 2 macroglobulin			
	e)	1108.49	BSQEHCYDR	no homologous			
3	a)	2090	EDTVQLQELTR	no homologous	emb|CAG05099.1 [Tetraodon nigroviridis]	185	Glycogen phosphorylase
	b)	1830.98	LSLPVSVDLAPTAR	no homologous			
	c)	1215.71	BLGQWLNVETR	no homologous			
	d)	1566.78	BDFNVGGYLQAVLDR	Glycogen phosphorylase			
	e)	1355.76	BDYYFALAHAER	Glycogen phosphorylase			
4	a)	2096.11	ENDDPXXDLSXXVR	Alpha-fetoprotein/serum albumin	emb|CAK18227.1 [Eublepharis macularius]	259	Transferrin
	b)	2075.99	BLSPSALVEACSFHQDACAR	Transferrin	sp|P84407.1 FETA_CHICK	131	Alpha-fetoprotein/serum albumin
	c)	1999.88	BCSSPSQELYYGYTGAFR	Transferrin			
	d)	1675.89	BTDDLWNLLSTVQDR	Transferrin			
	e)	1910.84	QFAENHDG	no homologous			
	f)	1056.55	BSSAGWNLPVGR	Transferrin			
	g)	1492.87	BRHPEFSPQLLLR	Alpha-fetoprotein/serum albumin			
5	a)	1910.91	BGXXXXXFAENHDGHXXR	Serum albumin/Alpha-fetoprotein	gb|AAM46105.1 [Hoplodactylus maculatus]	218	Serum albumin/Alpha-fetoprotein
	b)	1593.72	BVCQCCSNSYAFR	Serum albumin/Alpha-fetoprotein	ref|XP_002944396.1 [Xenopus (Silurana) tropicalis]	67	Hemopexin-like
	c)	1742.83	BCXXXPFQAFSSDNAGR	Hemopexin-like	ref|XP_002415879.1 [Ixodes scapularis]	70	Esterase
	d)	1492.83	BRHPEFSPQLLLR	Serum albumin/Alpha-fetoprotein			
	e)	1485.79	BSGSPVYAYTFTHR	Esterase			
	f)	1356.85	BRVWCMAHNALR	no homologous			
	g)	1199.65	BFDPVTATVPPR	no homologous			
	h)	1068.58	BNECLLSYR	no homologous			
6	a)	1900.79	BNYCDNPGEYWLGNER	fibrinogen	ref|XP_002752372.1 [Callithrix jacchus]	203	Pregnancy-zone protein /a2M/Ovostatin/a1M/murine globulin/a1-proteinase inhibitor 3
	b)	1822.83	BSCTFYEFTNQLDQR	Nesprin-2 like	gb|ACF32095.1 [Megapodius eremita]	113	Fibrinogen beta chain/Angiopoitin-2
	c)	1742.97	BLWTVLSHLEXXVXXR	Alpha 2 macroglobulin	ref|XP_002933762.1 [Xenopus (Silurana) tropicalis]	65	Nesprin-2 like
	d)	1422.8	BNLSGNDVQHHFR	no homologous			
	e)	1513.78	BTPDVQLLHSSXXXK	no homologous			
	f)	1324.65	BHPDGSYSTFGTR	Alpha 2 macroglobulin,			
	g)	1226.67	BALGYLVSGYQR	Alpha 2 macroglobulin			
7	a)	2171.93	BXXXSLMTTEDNWTLLQNR	Fribrinogen	ref|XP_002196458.1 [Taeniopygia guttata]	276	Fibrinogen beta chain
	b)	1900.79	BNYCDNPGEYWLGNER	Fibrinogen	ref|XP_002192134.1 [Taeniopygia guttata]	246	Pyrovate kinase
	c)	1822.83	BSCTFYEFTNQLDQR	Fibrinogen			
	d)	1653.72	BEPXXXAWAEDVDLR	Pyrovate kinase			
	e)	1373.68	BNTGLLCTLGPASR	Pyrovate kinase			
	f)	1253.57	EDAGGWWYNR	Fibrinogen			
	g)	1043.56	BEAEAALFHR	Pyrovate kinase			
8	a)	2304.06	LSPNDQTEFF	Fibrinogen	ref|NP_001080639.1 [Xenopus laevis]	235	Fibrinogen gamma chain
	b)	2089.93	BCQQPCEDTVQLQELTGR	Fibrinogen			
	c)	1713.02	BLHLLTTQSTVPYALR	Fibrinogen			
	d)	1215.69	BRSDLLDEVLR	no homologous			
9	a)	1804.96	SPGALGGTTGLYEALELR	Alpha-enolase	gb|EDL27268.1 [Mus musculus]	72	Alpha-enolase
10	a)	1657.85	TFLVEGVSSLHAGGQR	no homologous	gb|AAF76899.1 [Zaocys dhumnades]	93	Creatine kinase
	b)	2050.94	RATTGTEAVQVFDLSNADR	Creatine kinase			
	c)	1785.95	CKDVVLKVAGFVDPPK	no homologus			
11	a)	2271.94	BRVPLAGTDGETTTQGLDGLSER	Fructose bisphosphate aldolase	gb|ABF60005.1 [Polypterus senegalus]	266	Fructose bisphosphate aldolase
	b)	2130.18	BLTPTTPSHLALLENANVLAR	Fructose bisphosphate aldolase			
12	a)	1765.77	BLVSWYDNEYGYSNR	GAPDH	ref|NP_989636.1 [Gallus gallus]	281	glyceraldehyde-3-phosphate dehydrogenase (GAPDH)
	b)	1611.93	BLVLDGHALTLFXXR	GAPDH			
	c)	1570.81	BVPTXXVSVVDLTCR	GAPDH			
	d)	1443.85	BDLLHALEPLVAPR	no homologous			
13	a)	1567.81	BDVGVTWVLLGHSER	Triosephosphate isomerase B	ref|NP_001133174.1 [Salmo salar]	120	triosephosphate isomerase B
	b)	1505.72	BEAVAQLDASNFGQR	Triosephosphate isomerase B			
	c)	1318.71	BTLVQEQLANFR	no homologous			
14	a)	2205.13	BFSVNLGQNDSNFVLHFNPR	Galectin	ref|NP_002299.2 [Homo sapiens]	198	Galectin-1 / Galectin-9
	b)	1495.74	BFDEDGGVLVCNTR	Galectin			
	c)	1136.53	BVAGQWGSEM(OX)R	Galectin			
	d)	1120.55	BVAGQWGSEMR	Galectin			
	e)	973.56	BVVAHALAHR	no homologous			
15	a)	2205.13	BFSVNLGQNDSNFVLHFNPR	Galectin	ref|NP_002299.2 [Homo sapiens]	198	Galectin-1 / Galectin-9
	b)	1885.83	BTYFPHYDMAAGSEQLR	Hemoglobin	gb|ABN71134.1 [Peromyscus maniculatus]	99	hemoglobin alpha subunit 1
	c)	1495.74	BFDEDGGVLVCNTR	Galectin			
	e)	1136.53	BVAGQWGSEM(OX)R	Galectin			
	f)	1120.55	BVAGQWGSEMR	Galectin			
	g)	973.56	BVVAHALAHR	Hemoglobin			
